# The Gut–Brain Axis in Post-Traumatic Stress Disorder: From Biological Mechanisms to Microbiome-Based Therapeutic Strategies—A Narrative Review

**DOI:** 10.3390/microorganisms14051091

**Published:** 2026-05-11

**Authors:** Eun Jin Yang, Hee Ra Park

**Affiliations:** NeuroGlymph Imaging and Modulation Center (NIMC), Korea Institute of Oriental Medicine (KIOM), Daejeon 34054, Republic of Korea

**Keywords:** post-traumatic stress disorder, gut microbiota, brain–gut–microbiota axis, microbiome-based intervention

## Abstract

Post-traumatic stress disorder (PTSD) is a debilitating psychiatric condition that impairs psychological functioning and increases susceptibility to various chronic illnesses, including inflammatory, metabolic, and cognitive disorders. Recent advances in neuroscience and microbiology have identified the brain–gut–microbiota axis as a key mediator of neuroimmune and neuroendocrine regulations, providing new insight into the pathophysiology of PTSD. This review synthesizes current findings from preclinical and clinical studies on gut microbiome alterations in PTSD, highlighting the underlying mechanistic pathways. Dysbiosis in PTSD is associated with immune dysregulation, altered neuroendocrine signaling, and neurotransmitter imbalances. Animal models, particularly those using the single prolonged stress paradigm, have demonstrated behavioral and microbial changes that mirror the characteristics of human PTSD. Human studies have revealed reduced abundance of beneficial bacterial taxa and increased inflammation-associated genera in patients with PTSD. Although emerging evidence supports the role of gut microbiota in PTSD, further research is needed to establish causal relationships and optimize microbiome-targeted therapies. Overall, the gut microbiome offers a novel and potentially modifiable target for the prevention and treatment of PTSD.

## 1. Introduction

Despite considerable progress in understanding the prevalence, symptomatology, and long-term consequences of post-traumatic stress disorder (PTSD), critical questions remain regarding the sources of inter-individual variability in vulnerability and the mechanisms linking PTSD to chronic disease comorbidities. These differences may arise as a consequence of chronic psychological stress and the downstream effects of cortisol and other stress-related hormones, which are known to modulate microbial composition and immune function [1]. Emerging evidence suggests that the gut microbiome may represent a potential mediator linking stress exposure to immune and neurobiological alterations. Both stress-induced hormonal signaling and pre-existing host factors may influence microbial composition, while microbiome alterations themselves may modify stress responsiveness. This suggests a bidirectional relationship between PTSD and gut microbiome [2,3], which remains relatively underexplored.

The human gastrointestinal (GI) tract harbors trillions of microorganisms, collectively referred to as the gut microbiota, whereas the microbiome includes the microorganisms and their genomes, metabolites, and environmental interactions within a defined ecosystem [4]. These commensal microbes are not mere bystanders; rather, they play essential roles in immune regulation, metabolic function, inflammation, and neurodevelopment [5,6]. Emerging evidence implicates gut microbiota dysbiosis—an imbalance within the microbial ecosystem—in the pathophysiology of neuropsychiatric disorders, including anxiety and depression [6,7]. The brain–gut–microbiota axis represents an intricate, bidirectional communication network linking the gut microbiota with the central nervous system (CNS) via endocrine, immune, metabolic, and neural pathways [8]. Psychological stressors, such as those observed in PTSD, can alter gut microbiota composition via the activation of limbic system structures (e.g., amygdala, hippocampus, and prefrontal cortex), thereby promoting the subsequent release of stress mediators, including noradrenaline [7].

The gut–brain axis, particularly the contribution of the gut microbiome, has emerged as a promising area of investigation in mental health research. Increasing evidence suggests that alterations in gut microbiota composition are associated with various psychiatric disorders, including depression, anxiety, and bipolar disorder [9,10,11]. This has opened new avenues for microbiome-targeted interventions, such as dietary modifications, probiotics (live beneficial microorganisms), and prebiotics (non-digestible compounds that promote microbial growth). Despite the nascent stage of related research, recent findings support the therapeutic potential of these strategies. A randomized controlled trial reported reductions in stress and anxiety following a high-prebiotic diet, along with enhanced psychological well-being associated with probiotic supplementation compared with placebo [12]. The gut microbiota has been shown to modulate brain function and behavior via several mechanisms, including microbial metabolite signaling (e.g., gamma-aminobutyric acid (GABA), short-chain fatty acids (SCFAs), and serotonin precursors), cytokine release by mucosal immune cells, and stimulation of afferent fibers of the vagus nerve [6,8,13]. Importantly, human studies have produced direct evidence linking gut microbiome alterations to PTSD. Specifically, decreased abundance of SCFA-producing bacteria, such as members of the *Ruminococcaceae* and *Lachnospiraceae* families, and increased abundance of potentially pro-inflammatory genera, including *Odoribacter*, *Catenibacterium*, and *Veillonella*, are associated with PTSD [14,15]. These microbial alterations have been correlated with PTSD symptom severity and systemic inflammatory markers, suggesting a mechanistic link between gut dysbiosis, immune activation, and neurobiological dysfunction in PTSD. Maintaining homeostasis across the GI, immune, and nervous systems is increasingly recognized as a critical aspect for supporting mental health [8]. Recent studies suggest that the gut microbiome mediates the link between systemic inflammation and stress-related neurobiological processes [6,16]. However, the specific roles of the gut microbiome in the pathophysiology of PTSD remain poorly understood and warrant further investigation.

This review synthesizes current preclinical and clinical evidence linking gut microbiota alterations to PTSD, focusing on interventional studies that target microbial pathways. Specifically, we sought to explore how gut microbiota dysbiosis contributes to PTSD-related symptomatology and assess the therapeutic potential of gut microbiome-modulating strategies.

## 2. Potential Mechanisms Related to the Gut–Brain Axis in PTSD

The hypothalamic–pituitary–adrenal (HPA) axis is the central regulator of the physiological response to stress, and its dysregulation is a well-documented characteristic of PTSD [17]. Cortisol, which is the end product of the HPA axis activation, exerts potent immunosuppressive and anti-inflammatory effects. Pro-inflammatory cytokines, such as interleukin 6 (IL-6) and tumor necrosis factor-alpha (TNF-α), can stimulate the HPA axis activity, reflecting a bidirectional relationship between neuroendocrine and immune systems [17]. Accumulating evidence suggests that the gut microbiome influences the development, calibration, and responsiveness of the HPA axis, thereby modulating central stress reactivity [18]. This crosstalk provides a plausible biological mechanism through which gut microbiota dysbiosis may contribute to PTSD pathogenesis and progression.

Several recent reviews have contributed to elucidating this emerging area of research. Leclercq et al. [19] proposed that the gut microbiome may shape individual vulnerability to PTSD following trauma exposure. Brenner et al. [20] conducted a systematic review on prebiotic and probiotic interventions in individuals with traumatic brain injury and PTSD, reporting preliminary evidence of therapeutic potential. Additionally, Malan-Muller et al. [21] emphasized the gut microbiome’s relevance in psychiatric disorders, particularly in anxiety- and trauma-related conditions, and advocated for its consideration as a therapeutic target. Eric et al. have further explored the reciprocal influence of trauma on gut microbiota composition and the role of the brain-gut-microbiota axis in modulating neuropsychiatric resilience or susceptibility [22].

**Figure 1:**
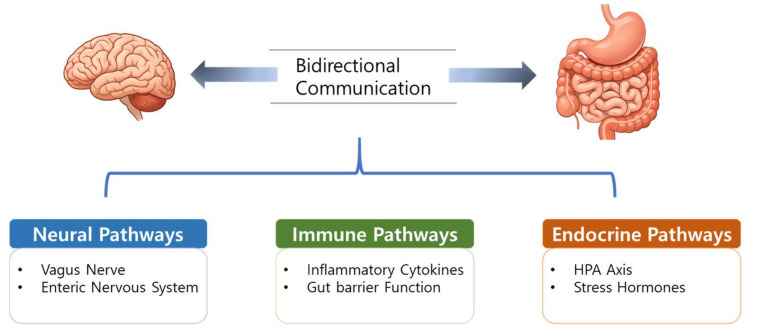
Overview of the gut–brain axis. The gut–brain axis is a bidirectional communication network linking the gut microbiota and the central nervous system through neural, immune, and endocrine pathways. These interconnected mechanisms influence stress responses and may contribute to the pathophysiology of PTSD. HPA, hypothalamic–pituitary–adrenal axis.

The bidirectional communication between the CNS and GI tract, commonly known as the gut–brain axis, is mediated through a multifaceted network involving the vagus nerve, sympathetic nervous system (via prevertebral ganglia), endocrine signaling, immune pathways, and humoral factors (Figure 1). A key component of this system is the gut microbiota, which links emotional and cognitive brain functions to GI activity [23]. The enteric nervous system (ENS) and vagus nerve have been highlighted as key neuronal pathways, as they mediate bottom-up gut–brain signaling that influences central stress- and fear-related circuits implicated in PTSD [24,25]. Gut-derived hormones, such as ghrelin, can further modulate vagal afferent activity and HPA axis responsiveness, suggesting a potential mechanistic link between altered gut signaling and dysregulated stress responses in PTSD [26,27]. The ENS, usually termed the “second brain,” contains more neurons than the spinal cord and synthesizes over 30 neurotransmitters. It releases neuroactive peptides and hormones, including ghrelin, that cross the blood–brain barrier (BBB) and act in concert with vagal afferents to influence processes such as appetite regulation [28].

### 2.1. Neuronal Pathway: Role of the Vagus Nerve in the Gut–Brain Axis of PTSD

The GI tract serves as a critical immune organ, while the vagus nerve exerts potent immunomodulatory effects, further reinforcing this pathway’s relevance in inflammation-related conditions [29]. Vagus nerve stimulation (VNS) and mind–body interventions, such as meditation, have demonstrated efficacy in alleviating symptoms of mood and anxiety disorders [30,31,32], as well as in conditions characterized by chronic inflammation [30]. Gut-directed hypnotherapy, a form of hypnotherapy specifically designed to modulate gut–brain interactions through symptom-focused suggestions, has demonstrated clinical benefits in patients with irritable bowel syndrome and inflammatory bowel disease (IBD), supporting the therapeutic relevance of targeting the gut–brain axis.

The vagus nerve is a key mediator linking dietary factors with neurological, psychiatric, and inflammatory processes. Its ability to integrate immune, metabolic, and neural signals highlights its central role in the pathophysiology and treatment of gut–brain axis-related diseases. Microbiota-derived metabolites may influence vagal nerve activity both indirectly, via absorption through enterocytes and systemic circulation, and locally through interactions with gut epithelial and enteroendocrine cells that interface with vagal afferents [24]. This axis operates through multiple physiological pathways—including neural (e.g., vagus nerve), endocrine (e.g., HPA axis), and immune mechanisms—allowing reciprocal interactions among the brain, GI tract, and microbiota [33]. The vagus nerve transmits afferent signals to the nucleus tractus solitarius upon stimulation [34], which subsequently sends signals via projections to the amygdala and hypothalamus—the key regions involved in emotional and stress regulation. VNS enhances norepinephrine (NE) release within the basolateral amygdala [35], as well as in the hippocampus and cortex [36], and thereby facilitates extinction learning. This aligns with findings indicating that NE infusion into the amygdala improves fear extinction [37]. Pairing extinction training with VNS in animal models can induce the remission of fear responses and alleviate PTSD-like symptoms [38]. Mechanistically, VNS strengthens synaptic plasticity between the infralimbic medial prefrontal cortex and basolateral amygdala complex, thereby promoting conditioned fear extinction [39]. Furthermore, VNS potentially enhances extinction learning by suppressing sympathetic nervous system activity [40] and thereby reduces anxiety-driven physiological arousal and disruption of the conditioned stimulus–fear association. Importantly, a reduction in hippocampal activity, likely mediated by enhanced GABAergic signaling [41], is one of the consistent neurophysiological outcomes of VNS. Considering the central role of the hippocampus in contextual memory and emotional regulation within the fear circuit, decreased hippocampal hyperactivity may contribute to reduced anxiety and improved emotional stability. This VNS-induced modulation of hippocampal function has also been observed in other psychiatric disorders, such as depression [42,43] and schizophrenia [44], further underscoring its potential to normalize dysregulated neural activity across affective and cognitive domains. Collectively, these findings position the vagus nerve as a pivotal modulator within the gut–brain axis that influences both neural and autonomic pathways that are implicated in PTSD pathophysiology.

Stress-induced CNS activation has been shown to influence gut microbiota composition, GI motility, intestinal permeability, and luminal neurotransmitter secretion. Non-microbial interventions, such as VNS and behavioral conditioning, may indirectly influence the gut microbiome through alterations in autonomic tone and stress-related neuroendocrine signaling (e.g., HPA axis activity), potentially affecting microbial composition, gastrointestinal motility, and barrier function. Conversely, bottom-up signaling originating from the gut microbiota affects stress-related neurocircuitry and behavior in animal models via neuroimmune and neuroendocrine pathways [24,45,46]. Gut microbiota composition plays a role in modulating key CNS functions, such as neuronal excitability and fear extinction learning, which are highly relevant to anxiety-related disorders [47].

Several studies have reported beneficial effects of prebiotic supplementation (i.e., dietary fibers that promote the growth of beneficial microorganisms) [46,48] and probiotic administration (i.e., live microbiomes with therapeutic potential) on mood, cognitive function, and stress-related symptoms [49]. In related anxiety disorders, probiotic strains such as *Bifidobacterium* have shown modest improvements in anxiety symptoms., although effects are variable [50]. Although these findings do not directly demonstrate therapeutic efficacy in PTSD, they suggest that gut microbiome–targeted interventions hold potential relevance for stress-related psychiatric disorders. Existing human and animal studies specifically investigating PTSD and the gut microbiome remain limited and preliminary, underscoring the need for rigorous, PTSD-focused clinical investigations [51].

### 2.2. Immunological Pathways in the Gut–Brain Axis of PTSD

The human immune system constitutes a complex and highly coordinated network that involves both innate and adaptive components, functioning as a critical defense mechanism against pathogenic insults while preserving internal physiological balance [52]. Notably, the gut microbiome plays a fundamental role in shaping host immune responses by regulating the maturation of innate and adaptive immune pathways, including T cell differentiation and cytokine signaling. Disruptions in microbiome–immune interactions may promote chronic low-grade inflammation and altered immune reactivity, which have been implicated in increased stress sensitivity and vulnerability to PTSD [24]. Consistent with this framework, recent studies have increasingly implicated both innate and adaptive immune responses in PTSD pathophysiology [53,54,55]. Clinical and epidemiological evidence suggests that PTSD is associated with altered immune regulation, characterized by chronically elevated low-grade inflammatory markers, including C-reactive protein (CRP), interferon-gamma (IFN-γ), IL-6, IL-10, and TNF-α [56,57,58]. In addition to this persistent inflammatory state, acute increases in pro-inflammatory cytokines during stress exposure, trauma recollection, or heightened anxiety have been reported. Collectively, these findings suggest both baseline immune dysregulation and stress-reactive immune activation in individuals with PTSD. Although multiple meta-analyses and systematic reviews have investigated immune alterations in PTSD, the findings remain heterogeneous and partially inconsistent. For example, Yang and Jiang [57] reported that several commonly studied pro-inflammatory markers were elevated in PTSD, whereas others, including soluble IL-2 receptor, were not consistently altered. This variability suggests that while a general pattern of low-grade inflammation is frequently observed in PTSD, not all immune markers are uniformly altered, highlighting the complexity and heterogeneity of immune dysregulation in PTSD pathophysiology. Passos et al. [58] identified substantial heterogeneity among studies assessing IL-1β, IL-6, and CRP and reported evidence of potential publication bias for IL-1β based on Egger’s regression test, suggesting that the magnitude of inflammatory alterations may have been overestimated in the literature. These findings underscore that immune dysregulation in PTSD is complex and not fully consistent across studies, warranting cautious interpretation and emphasizing the need for standardized, large-scale investigations. Crucially, the gut microbiome is recognized as a central regulator of systemic inflammation. Several commensal bacterial taxa, including *Roseburia* and *Odoribacter*, are known to exert anti-inflammatory effects primarily through the production of beneficial metabolites, such as SCFAs [51,59]. These metabolites influence immune cell differentiation, cytokine production, and intestinal barrier integrity—all of which are mechanisms with downstream effects on brain function and stress-related pathology.

Given the converging roles of the immune system and gut microbiota in modulating inflammatory responses, investigating their interplay in PTSD is essential for elucidating this disorder’s mechanistic underpinnings. Such insights may pave the way for novel microbiome- or immunomodulation-based therapeutic strategies aimed at mitigating PTSD symptoms and comorbid inflammatory conditions. Some gut microbiota species exert anti-inflammatory functions, and their depletion has been associated with GI inflammation [60,61,62]. Beneficial species, such as specific *Clostridium* strains, ferment carbohydrates into SCFAs, which have both local and systemic immunomodulatory functions. SCFAs support gut barrier maintenance by promoting anti-inflammatory cytokine release [63], enhancing mucus layer integrity [64], and activating regulatory T-cells [65]. Therefore, stress-induced dysbiosis may compromise intestinal barrier function and perpetuate a cycle of inflammation [66].

Commensal microbes also influence central immune processes, including microglial development and activity, through gut–brain axis pathways. Microglia in germ-free or antibiotic-treated mice have been shown to display impaired maturation and function, which can be restored through recolonization with a healthy microbiome or supplementation with SCFAs, particularly butyrate [67]. This gut–brain–immune interaction likely occurs through vagus nerve signaling and/or systemic circulation of microbial metabolites [68,69]. While the exact mechanisms remain partly understood, accumulating evidence suggests that stress-induced microbial alterations contribute to chronic low-grade systemic inflammation and neuroinflammation observed in mood disorders [70]. Further investigations are needed to clarify how disruptions in gut microbiota composition, gut barrier integrity, and gut–brain axis signaling contribute to stress-related pathophysiology. Emerging hypotheses suggest that both the BBB and intestinal epithelial barrier are vulnerable targets of stress-exacerbated inflammation.

In PTSD, repeated acute stress and persistent sympathetic nervous system hyperactivity may lead to impaired glucocorticoid signaling, resulting in dysregulated immune responses over time [55,71]. Evidence from animal models supports this mechanism. Chronic—but not acute—social defeat stress has been shown to decrease morning corticosterone levels in stress-susceptible mice [72]. Cortisol suppresses adaptive immune responses during acute stress to conserve resources for immediate survival, shifting immunity toward humoral pathways [73,74]. However, this immune shift can paradoxically lead to heightened inflammation when stress becomes prolonged [73]. Pro-inflammatory cytokines, such as IL-1β, IL-6, and TNF-α, can cross the BBB via specialized transport mechanisms [75]. Experimental investigations in mice have confirmed that peripherally administered IL-1α and IL-1β can be transported into the brain [76]. Nevertheless, cytokine transport is saturable, suggesting that a concurrent increase in BBB permeability is necessary to trigger pathological central immune activation.

### 2.3. Endocrine Pathway: The ENS and Neuroendocrine Signaling in PTSD

**Figure 2:**
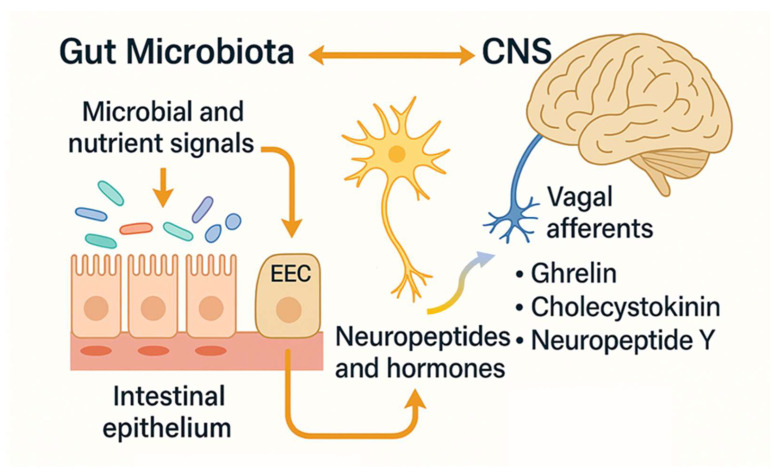
Gut–brain communication via enteroendocrine signaling. Bidirectional interaction between the gut microbiota and CNS through EECs. EECs located in the intestinal epithelium function as chemosensors that detect luminal microbial and nutrient signals via toll-like receptors and other molecular sensors. Upon activation, they release neuropeptides and hormones, such as ghrelin, cholecystokinin, and neuropeptide Y, which modulate gastrointestinal motility and appetite, as well as signal to the CNS via vagal afferents or by crossing the blood–brain barrier. Sensory information from the gut is transmitted to the brain via vagal afferent fibers, providing a rapid neural route for gut–brain communication. In parallel, circulating neuropeptides and hormones exert endocrine effects on the CNS, modulating stress responsiveness, emotional regulation, and autonomic function. Dysregulation of these interconnected pathways, driven by gut microbial imbalance and altered neuroendocrine signaling, may contribute to chronic inflammation, neuroimmune activation, and maladaptive stress responses observed in post-traumatic stress disorder. These pathways contribute to the regulation of emotion, stress responses, and memory, underscoring the role of EECs in gut–brain axis communication. CNS, central nervous system; EEC, enteroendocrine cell.

The gut microbiota communicates with the CNS not only through neural and immune mechanisms but also via gut endocrine pathways [77]. A key interface in this communication is formed by enteroendocrine cells (EECs), which are specialized sensory cells in the intestinal lining that can detect luminal contents and interact with vagal afferents through chemosensory mechanisms [78]. These cells orchestrate physiological responses to nutrients, such as carbohydrates, triglycerides, and proteins, by releasing hormones and peptides that influence GI motility, secretion, and host metabolic behavior [79]. EECs also serve as nutrient and microbial sensors. They express toll-like receptors (TLRs) and other microbial metabolite receptors, enabling them to recognize microbial signals and contribute to the regulation of gut functions, including motility and appetite [80,81]. The gut microbiota can modulate EEC activity to induce the release of neuroactive substances such as ghrelin, gastrin, orexin, galanin, cholecystokinin, leptin, and neuropeptide Y. These signaling molecules can affect peripheral neural communication via the vagus nerve and also reach the CNS to influence emotional and behavioral states [80] (Figure 2).

Accumulating evidence suggests that microbial signals influence subliminal interoceptive processing, thereby affecting memory encoding, emotional arousal, and affective states by modulating activity in brain regions, such as the insular cortex, anterior cingulate cortex, orbitofrontal cortex, and amygdala [82]. Overall, these findings underscore the integral role of gut microbiota-EEC interactions in shaping gut–brain communication and their potential relevance to stress-related disorders, such as PTSD. In addition to immune and neural routes, the gut microbiota communicates with the CNS via gut-derived endocrine pathways, including modulation of HPA axis and gut hormone secretion (e.g., Glucagon-like peptide-1, Peptide YY), which are directly or indirectly modulated by microbial metabolites, particularly short-chain fatty acids, via their interaction with enteroendocrine cell receptors.

## 3. Therapeutic Prospects of PTSD Based on the Gut–Brain Axis

**Figure 3:**
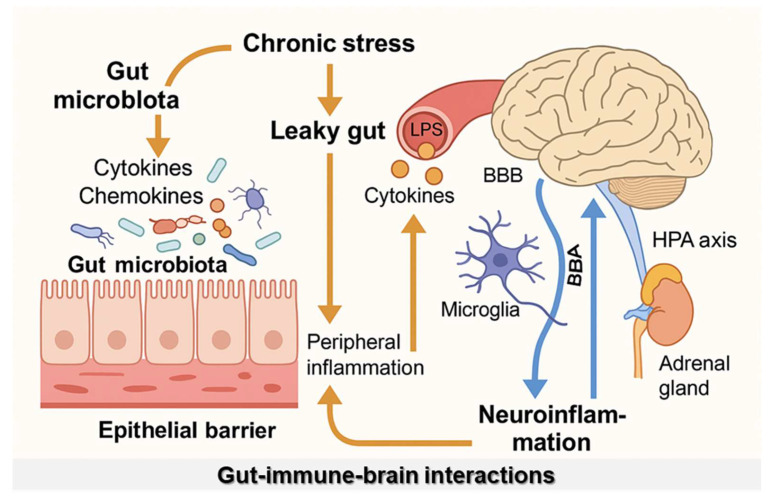
Dysregulation of the gut–immune–brain axis in response to chronic stress. Proposed mechanism by which chronic psychological stress induces gut microbiota dysbiosis and disrupts the intestinal epithelial barrier, leading to increased intestinal permeability (“leaky gut”). Translocation of microbial products, such as lipopolysaccharide, into the circulation promotes peripheral inflammation through elevated cytokine production. These inflammatory mediators can cross the BBB or signal via the vagus nerve, activating microglia and engaging the HPA axis, ultimately resulting in neuroinflammation. The figure highlights the complex interplay among the gut microbiota, immune signaling, and brain function in the pathophysiology of stress-related disorders, such as post-traumatic stress disorder. BBA, brain–body axis; BBB, blood–brain barrier; HPA, hypothalamic–pituitary–adrenal.

The gut microbiota contributes to a balanced basal inflammatory state by stimulating the controlled release of cytokines and chemokines under homeostatic conditions. The GI epithelial barrier—particularly its mucus layer—forms the primary interface for host–gut microbiota interactions, where innate immune mechanisms play a central role [83]. Intestinal epithelial cells—such as enterocytes—express pattern recognition receptors, including TLR, thereby enabling the recognition of microbial components—including lipopolysaccharide (LPS)—and facilitating the release of cytokines and chemokines that shape local immune responses [83,84]. However, pathological conditions—such as chronic psychological stress—result in increased intestinal permeability, a phenomenon commonly known as “leaky gut” [85]. This disruption facilitates the translocation of microbial products, such as LPS, into the bloodstream, triggering systemic inflammation. Increased intestinal permeability (“leaky gut”) is a mechanism proposed to link gut microbiome dysbiosis to PTSD-related neuroimmune changes. Microbial translocation can trigger systemic inflammatory responses, increasing circulating cytokines that influence brain function through vagal signaling, circulation, and BBB modulation, ultimately contributing to HPA axis dysregulation [86,87] (Figure 3). Nevertheless, as existing evidence is largely associative, the model presented in Figure 2 should be interpreted as a conceptual framework summarizing current hypotheses regarding brain–gut–microbiota interactions in PTSD rather than a definitively established mechanistic pathway.

Notably, compromised BBB function allows peripheral cytokines, including IL-1 and IL-6, to enter the CNS and activate neuroendocrine structures, such as the hypothalamus and circumventricular organs [88]. Animal studies further demonstrated that the gut microbiota is essential for the maintenance of BBB integrity and the development and maturation of microglial cells—central immune cells of the brain [67,89]. Collectively, these findings highlight the role of gut–immune–brain interactions in PTSD, in which trauma-associated gut microbiota dysbiosis may contribute to chronic low-grade systemic inflammation, neuroimmune activation, and dysregulation of the HPA axis, ultimately influencing stress reactivity and PTSD symptom persistence. Emerging evidence increasingly implicates the brain–gut–microbiota axis in PTSD-related symptomatology. Gut microbiome dysbiosis, characterized by reduced microbial diversity and altered abundance of key commensal taxa, has been associated with impaired intestinal barrier function and increased inflammatory signaling. These changes may contribute to stress-related immune activation and neuroinflammatory processes relevant to PTSD. Findings from a previous meta-analysis revealed that individuals with PTSD may exhibit a relatively reduced abundance of beneficial bacterial phyla, such as *Actinomycetota*, *Lentisphaerae*, and *Verrucomicrobia*, whereas the abundance of pro-inflammatory taxa, including *Enterococcus, Escherichia*, and *Shigella*, may be increased [90]. However, it should be noted that gut microbiome profiles are highly influenced by factors such as population characteristics, diet, medication use, comorbidities, and analytical pipelines.

### 3.1. Preclinical Paradigms for PTSD: A Gateway to Mechanistic Insights into the Gut–Brain Axis

Animal models are essential experimental tools for investigating mechanistic interactions between stress exposure, neurobiological alterations, and gut microbiota composition in PTSD-related research. Although modeling the heterogeneous and complex phenotype of PTSD in animals is challenging [91], rodent models remain indispensable tools for investigating stress-related neurobiology and gut–brain interactions [92]. Rodents exhibit evolutionarily conserved behavioral and neuroendocrine responses to threat, making them suitable for examining the neural circuitry implicated in PTSD [93]. A variety of experimental paradigms have been developed to model distinct aspects of trauma-related pathology, including physical stressors (e.g., foot shock and restraint), social stressors (e.g., maternal separation and social defeat), and psychological stressors (e.g., predator exposure) [94]. Each of these models captures distinct aspects of trauma-induced behavioral and neurobiological alterations. Although no single paradigm recapitulates the full PTSD phenotype, they enable the exploration of specific mechanistic questions, such as changes in synaptic plasticity, HPA axis function, immune signaling, and gut microbiome composition. Current data on the relationship between gut microbiota composition, microbial diversity, and PTSD-related symptomatology remain limited.

Zhou et al. [95] employed the SPS model to assess the correlations among gut microbiota, behavior, and neurotransmitter levels in rats. Their findings indicated that single prolonged stress (SPS)-exposed rats exhibited pronounced fear-related behaviors, such as increased freezing time in fear-conditioning tasks and reduced exploratory behavior in the open field test—both indicative of heightened anxiety states. Significant alterations in gut microbiota composition have been observed in SPS model rats compared with controls. Taxonomic analysis revealed changes across the order, family, and genus levels, with particular changes in the relative abundances of *Firmicutes*, *Bacteroidetes*, *Cyanobacteria*, and *Proteobacteria* [95]. These microbial signatures were associated with fear- and anxiety-like behaviors, as well as reductions in brain serotonin concentrations. Complementary findings in the hippocampus and prefrontal cortex further validate the neurobiological relevance of the SPS model in replicating PTSD-associated neurocircuitry and neurotransmission alterations [95]. Collectively, these findings underscore the utility of the SPS model in elucidating the bidirectional relationship between gut microbiota and neurobehavioral responses to trauma. However, it is important to acknowledge that the SPS paradigm cannot fully reproduce the complexity of human PTSD, particularly symptoms such as intrusive memories and cognitive distortions. Moreover, SPS models may not adequately capture clinically relevant dimensions, including sex differences, developmental trauma exposure, and the chronic and heterogeneous nature of PTSD. Therefore, findings derived from SPS models should be interpreted with caution and considered as only partially representative of the disorder.

In addition to commonly used stress paradigms, the chronic subordinate colony stress (CSCS) model has been increasingly utilized to investigate PTSD-like phenotypes. This model is based on repeated social defeat and chronic psychosocial stress exposure and closely mimics key features of PTSD, including heightened anxiety, social avoidance, and dysregulated stress responses [96,97]. Importantly, CSCS has been shown to induce alterations in gut microbiota composition, increased intestinal permeability, and enhanced systemic inflammation, supporting its relevance in investigating microbiota–gut–brain axis interactions in stress-related disorders [98,99].

### 3.2. Gut Microbiota Alterations in PTSD: Evidence from Human Clinical Studies

Human clinical studies have examined gut microbiome alterations across diverse populations affected by PTSD. Evidence from translational and human research suggests that stress-induced dysbiosis, particularly during critical developmental periods such as early life, may lead to long-term alterations in immune regulation, intestinal barrier integrity, and neuroendocrine signaling, thereby increasing vulnerability to stress-related disorders. A notable example comes from a South African cohort with high exposure to trauma and interpersonal violence, in which gut microbiome composition differed between individuals with PTSD and trauma-exposed controls: individuals with PTSD showed reduced relative abundance of several commensal taxa, including *Actinomycetota, Lentisphaerae*, and *Verrucomicrobia* [3]. These microbial alterations may have functional relevance, as many commensal bacteria contribute to the production of SCFAs, maintenance of epithelial barrier integrity, and modulation of inflammatory signaling pathways implicated in stress sensitivity and neuroimmune communication. Consistent with this framework, epidemiological studies have demonstrated an association between PTSD and GI disorders, including IBD [100]. This relationship appears bidirectional, as IBD is associated with an increased PTSD risk, while PTSD has been linked to worsened IBD symptom severity [101]. Gut microbiome dysregulation has also been implicated in inflammation-associated conditions such as IBD [102], cardiometabolic disorders [103], and diabetes [104], suggesting shared immune and metabolic pathways. However, a recent meta-analysis examining associations between the gut microbiome and psychiatric disorders was unable to generate evidence specific to PTSD owing to the limited number of targeted studies [105]. To date, only a few observational human studies have investigated the PTSD–gut microbiome relationship. An early pilot study conducted in a South African cohort (*n* = 30) found that three bacterial phyla (*Actinomycetota*, *Lentisphaerae*, and *Verrucomicrobia*) were differentially abundant in individuals with PTSD compared with trauma-exposed controls without PTSD, and their relative abundance negatively correlated with PTSD severity [30]. Another study identified a reduction in microbial diversity and beneficial taxa (e.g., *Lachnospiraceae* and *Ruminococcaceae*) in combat-exposed veterans with cirrhosis (*n* = 93) and an increase in pathobionts (e.g., *Enterococcus*, *Escherichia*, and *Shigella*) in those with PTSD [106].

Regarding therapeutic interventions, preliminary clinical evidence on microbiome-targeted approaches in PTSD remains limited. An early pilot study in 10 combat veterans reported that 6 months of supplementation with a fermented soy formulation (FSWW08) (administered daily) was associated with improvements in anxiety, detachment, and several somatic symptoms [107]. However, direct evidence demonstrating that FSWW08 alters gut microbiome composition remains limited, and its effects on microbial profiles have not been fully characterized. Although the intervention is presumed to influence gut microbial activity, direct measurements of microbiome composition or microbial metabolites were limited, and interpretation of these findings is constrained by methodological limitations, including small sample size, absence of a placebo-controlled design, and heterogeneity in PTSD diagnostic criteria [107]. In a randomized, placebo-controlled trial (*n* = 31), supplementation with *Lactobacillus reuteri* DSM 17,938 (1 × 10^8^ CFU/day for 8 weeks) was associated with a non-significant trend toward reduced plasma CRP levels and attenuated physiological stress responses compared with placebo, based on stress-response assessments [108]. As CRP is a non-specific inflammatory marker, these findings provide only indirect evidence linking microbiome modulation to PTSD-related physiological pathways.

An additional study demonstrated that PTSD in veterans is associated with gut microbiome dysbiosis, including reduced SCFA-producing bacteria, although these changes are strongly influenced by comorbid and environmental factors [109]. Another pilot randomized controlled trial suggested that prebiotic supplementation may modestly improve PTSD symptoms while inducing changes in gut microbiota composition [110].

Together, these preliminary studies provide suggestive evidence that the gut microbiome contributes to the expression and severity of PTSD symptoms; however, additional mechanistic and interventional research is needed to clarify causality and therapeutic potential. Table 1 summarizes representative human and translational studies examining gut microbiota alterations in PTSD. It highlights consistent associations with reduced microbial diversity, altered immune-related taxa, and PTSD symptom severity, while also underscoring substantial methodological heterogeneity and limitations.

### 3.3. Therapeutic Prospects of Gut Microbiota Modulation in PTSD

Recent studies have identified the gut microbiome as a significant contributor to PTSD pathophysiology [51]. Dysbiosis can disrupt immune and neurochemical regulation. Individuals with PTSD usually exhibit elevated levels of inflammatory markers (IL-6, IL-10, TNF-α, and IFN-γ), potentially mediated by microbial imbalance [111]. Commensal bacteria, such as *Roseburia* and *Odoribacter*, have been shown to exert anti-inflammatory effects via SCFAs, while other microbes influence neurotransmitter systems, including serotonin and GABA. Stress has been shown to alter microbial composition, creating a feedback loop that amplifies PTSD symptoms. Genetic studies suggest causal associations between specific bacterial genera (e.g., *Dorea* and *Sellimonas*) and PTSD [2]. Dietary patterns, particularly adherence to a Mediterranean diet, have been associated with symptom reduction and the enrichment of protective microbiota, such as *Eubacterium eligens* [112]. However, as several studies in this area are based on small sample sizes, their statistical power is limited; therefore, these findings should be regarded as exploratory and interpreted with caution. SCFAs have been shown to modulate neuroinflammation, stress responses, and gut–brain axis signaling [113,114]. Probiotics, particularly *Lactobacillus* and *Bifidobacterium* species, can influence microbial composition and contribute to SCFA production [115], and both preclinical and human studies have reported that probiotic interventions can alter emotional behavior and stress-related outcomes [116,117]. Together, these findings suggest a potential mechanistic link between probiotics, SCFA-mediated signaling, and PTSD-related pathophysiology, although direct clinical evidence remains limited.

Several probiotic-based interventions are under investigation and summarized in the main findings discussed above. In addition, microbiota-modulating interventions—such as probiotics, prebiotics, and certain antibiotics—have demonstrated potential benefits in reducing the symptoms of stress-related conditions, including anxiety and depression, in both preclinical models and patients with disorders such as irritable bowel syndrome or chronic fatigue syndrome [118]. Notably, a preclinical study demonstrated that the administration of heat-killed *Mycobacterium vaccae*, an immunoregulatory environmental microbe, attenuated PTSD-like symptoms and promoted an anti-inflammatory immune profile [119]. In this study, heat-killed *Mycobacterium vaccae* (approximately 10^7^ CFU equivalent) was administered subcutaneously prior to stress exposure, and treated animals exhibited significant reductions in anxiety- and fear-related behaviors, including decreased fear conditioning responses, compared to controls. These findings suggest that gut microbiota modulation may influence stress-related phenotypes, although their translational relevance to PTSD remains to be fully established. Overall, while the gut microbiome represents a promising therapeutic target, current evidence is preliminary and insufficient to support clinical application in PTSD. In the future, well-designed, adequately powered clinical trials with clearly defined diagnostic criteria and validated outcome measures should be prioritized to determine the efficacy and specificity of microbiome-based interventions in PTSD.

## 4. Conclusions and Limitations

Despite recent advances in understanding the brain-gut-microbiota axis in PTSD pathophysiology, some limitations should be recognized. A limitation of the current literature is that the reported links between the gut microbiota and PTSD-related biological processes are primarily based on associative findings from animal and observational human studies. Consequently, the mechanistic pathways described in this review should be considered hypothesis-generating, as direct causal relationships remain to be clearly established. Most existing research comprises small, cross-sectional, or observational studies, with restricted ability to determine causal links between alterations in gut microbiota communities and PTSD symptoms. The lack of large-scale, longitudinal investigations involving well-characterized PTSD cohorts further limits the robustness and generalizability of the current findings. Most human studies investigating the association between gut microbiota and PTSD are cross-sectional or case-control in design, limiting causal inference regarding whether microbial dysbiosis precedes PTSD onset or occurs as a consequence of the disorder [5,120]. Observed gut microbiota alterations in PTSD populations may also reflect secondary effects of chronic stress exposure, dietary changes, sleep disturbances, or psychotropic medication use rather than a primary etiological role [24]. Preclinical evidence from the SPS model demonstrates that fecal microbiota transplantation (FMT) can partially reverse fear extinction deficits and normalize stress-related neuroendocrine alterations, supporting a functional contribution of gut microbiota to PTSD-like phenotypes [121]. However, to date, randomized controlled FMT trials in patients with PTSD are lacking, and direct causal evidence in humans remains extremely limited [122]. More recently, Mendelian randomization analyses have suggested that specific gut microbial taxa have potential causal effects on PTSD risk, offering a genetic approach to reduce confounding and reverse causation inherent in observational studies [2]. Nevertheless, the modest heritability of microbiome traits and the risk of horizontal pleiotropy constrain the robustness of these findings and warrant cautious interpretation [2]. Overall, existing evidence supports a contributory—but not yet definitive—causal role of gut microbiota dysbiosis in PTSD pathophysiology, highlighting the need for longitudinal studies and randomized microbiome-targeted interventions to establish causality [120,123]. A major limitation of current PTSD–gut microbiome studies is the potential confounding effect of psychotropic medications, as antidepressants, antipsychotics, and mood stabilizers may independently alter gut microbial composition, diversity, and metabolic activity through both direct effects on the microbiome and indirect effects mediated by metabolic changes, such as dyslipidemia, weight gain, and metabolic syndrome-like alterations, particularly associated with second-generation antipsychotics [124,125]. These medications may influence microbial taxa involved in SCFA production, bile acid metabolism, and neurotransmitter-related pathways, thereby potentially modifying immune regulation and stress-response signaling relevant to PTSD pathophysiology. In turn, microbiome alterations may also affect drug metabolism and therapeutic responsiveness, further complicating the interpretation of microbiome–PTSD associations. Given the high prevalence of long-term pharmacological treatment in PTSD populations, future studies should carefully control for medication use or prioritize drug-naive and longitudinal designs to better delineate disorder-specific microbiome alterations [6]. Significant methodological and demographic heterogeneity also persists across studies, including differences in sample populations, trauma exposure types, microbiome sequencing platforms, and analytical pipelines, all of which may influence the observed microbial profiles and limit cross-study comparability. Greater standardization of study design, metadata collection, and analytical methods will be important in elucidating reproducible microbiome signatures associated with PTSD.

Although preclinical models, such as the SPS paradigm, have provided valuable mechanistic insights, they fail to fully recapitulate the chronic, multifactorial nature of PTSD in humans. Moreover, gut microbiota composition is shaped by various confounding variables—including dietary habits, medication use, geographical factors, and lifestyle behaviors—that are not consistently controlled across studies. Although gut microbiome-based interventions, such as probiotics, prebiotics, or FMT, show preliminary promise, their therapeutic potential, long-term safety, and reproducibility are yet to be confirmed through rigorous randomized controlled trials. Overcoming these challenges will be critical for translating current experimental and clinical findings into reliable, evidence-based strategies for PTSD prevention and treatment.

**Figure 4:**
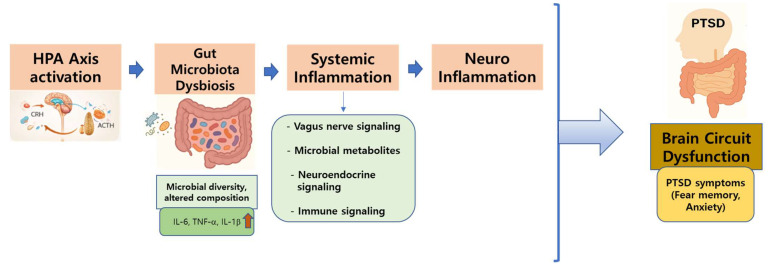
Gut–brain axis mechanisms linking traumatic stress to post-traumatic stress disorder-related brain dysfunction. Traumatic stress activates the HPA axis and contributes to gut microbiota dysbiosis, characterized by reduced microbial diversity and altered microbial composition. Gut microbiota dysbiosis promotes systemic inflammation and influences the brain through vagus nerve signaling, immune pathways, microbial metabolites (e.g., short-chain fatty acids, tryptophan metabolites, and gamma-aminobutyric acid), and neuroendocrine mechanisms. The integrated signals affect the amygdala, hippocampus, and prefrontal cortex, resulting in neural circuit dysfunction associated with fear memory and anxiety in post-traumatic stress disorder. HPA, hypothalamic-pituitary-adrenal; IL-1β, interleukin-1β; IL-6, interleukin-6; TNF-α, tumor necrosis factor-alpha.

The gut and CNS engage in continuous, bidirectional communication through neural (vagus nerve), immune, and endocrine pathways, all of which are profoundly shaped by the gut microbiota composition. Disruptions in this microbial balance or dysbiosis have been linked to immune system disturbances, dysregulated stress-hormone activity, increased BBB permeability, and altered synthesis of key neurotransmitters involved in emotional regulation in PTSD (Figure 4).

Preclinical research, particularly using the SPS model, has shown that trauma can induce significant shifts in gut microbiota communities, and this mirrors the behavioral and neurochemical abnormalities characteristic of PTSD. Similarly, clinical investigations in patients with PTSD have revealed a reduction in beneficial bacterial phyla, such as *Actinomycetota, Lentisphaerae*, and *Verrucomicrobia*, alongside an enrichment of pro-inflammatory genera, including *Enterococcus, Escherichia*, and *Shigella*. Emerging evidence suggests that modulating the gut microbiome can help mitigate PTSD symptoms. Interventions, such as adherence to anti-inflammatory dietary patterns (e.g., the Mediterranean diet); identification of protective microbial taxa, such as *Dorea* and *Sellimonas*; and probiotic supplementation, have demonstrated promising effects on emotional resilience and stress-related behaviors. Overall, these findings underscore the therapeutic potential of targeting the brain-gut-microbiota axis in PTSD management.

PTSD frequently co-occurs with other psychiatric disorders, including major depressive disorder and anxiety disorders, as well as substance use disorders, all of which may independently influence gut microbiome composition. Substance use (e.g., alcohol, nicotine, and other drugs) is particularly relevant, as it can significantly alter microbial diversity, intestinal permeability, and inflammatory responses. These factors represent important confounders that may complicate the interpretation of microbiome alterations observed in PTSD.

The gut microbiome is increasingly recognized as a key modulator of multiple systems involved in PTSD pathophysiology, including immune, neuroendocrine, and neural pathways. Alterations in microbial composition may influence inflammatory responses, HPA axis regulation, and neurotransmitter signaling. These interconnected mechanisms suggest a potential role for the gut microbiome in shaping stress responses and PTSD-related outcomes. However, further longitudinal and mechanistic studies are necessary to clarify causality and guide the development of microbiome-based interventions for PTSD.

## Figures and Tables

**Table 1:** Summary of representative human studies examining the relationship between PTSD and gut microbiota.

Study/Population	Sample Size	Microbiome Analysis	Main Findings	Key Limitations
South African trauma-exposed cohort [3]	*n* = 30	16S rRNA sequencing (fecal samples)	Individuals with PTSD showed significantly reduced abundance of *Actinomycetota, Lentisphaerae*, and *Verrucomicrobia* (adjusted *p* < 0.05); microbial abundance negatively correlated with PTSD severity.	Small sample size, cross-sectional design, and limited control for diet and medication
Combat-exposed veterans with cirrhosis [106]	*n* = 93	16S rRNA sequencing	PTSD was associated with reduced microbial diversity (Shannon index 2.1 ± 0.5 vs. 2.5 ± 0.5, *p* = 0.03), depletion of beneficial taxa (*Lachnospiraceae* and *Ruminococcaceae*), and enrichment of pathobionts (Enterococcus, Escherichia, and Shigella).	Comorbid liver disease, predominantly male cohort, and potential confounding by alcohol use
Fermented soy supplementation in combat veterans [107]	*n* = 10	Microbiome and symptom assessment	Six months of fermented soy intake was associated with reductions in anxiety, detachment, and somatic symptoms (descriptive change; statistical significance not consistently reported).	No control group, relatively small sample size, and placebo effect cannot be excluded
Lactobacillus reuteri DSM 17,938 trial [108]	*n* = 31	Plasma biomarkers + stress response measures	Probiotic supplementation showed trends toward reduced CRP levels and attenuated physiological stress responses (*p* > 0.05).	Underpowered study, no significant between-group differences, and short intervention duration
A United States-Veteran Microbiome Project (US-VMP) study (U.S. veterans with high PTSD prevalence) [109]	~300–700 (varies by sub-analysis)	16S rRNA gene sequencing	Microbiome composition varied significantly across clinical and environmental factors (PERMANOVA *p* < 0.05); however, PTSD was not independently associated with a distinct microbial signature.	Cross-sectional design (no causality). Strong confounding factors (diet, medication, comorbidities such as obesity and metabolic disease).
Prebiotics as an adjunct therapy for PTSD: a pilot randomized controlled trial (adults with PTSD) [110]	20–50 (pilot RCT)	16S rRNA sequencing	Prebiotic supplementation showed modest improvements in PTSD symptoms and stress-related measures; however, statistical significance was limited. Changes in microbiome composition were observed.	Small sample size (pilot study). Short intervention duration. Limited statistical power. SCFA/metabolite data were limited or indirect.

PTSD, post-traumatic stress disorder; RCT, randomized controlled trial; SCFA, short-chain fatty acid.

## Data Availability

No new data were generated or analyzed in this study.

## Abbreviations

The following abbreviations are used in this manuscript:

BBB	Blood–brain barrier
CNS	Central nervous system
CRP	C-reactive protein
CSCS	Chronic subordinate colony stress
EEC	Enteroendocrine cell
ENS	Enteric nervous system
GABA	Gamma-aminobutyric acid
GI	Gastrointestinal
HPA	Hypothalamic-pituitary-adrenal
IBD	Inflammatory bowel disease
IFN-γ	Interferon-gamma
IL-10	Interleukin-10
IL-1β	Interleukin-1β
IL-6	Interleukin-6
LPS	Lipopolysaccharide
NE	Norepinephrine
PTSD	Post-traumatic stress disorder
SCFA	Short-chain fatty acid
SPS	Single prolonged stress
TNF-α	Tumor necrosis factor-alpha
TLR	Toll-like receptor
VNS	Vagus nerve stimulation
